# Supplementary material for: The epidermis coordinates thermoresponsive growth through the phyB-PIF4-auxin pathway
Source: Nat Commun. 2020 Feb 26;11:1053. doi: 10.1038/s41467-020-14905-w (PMC7044213; doi:10.1038/s41467-020-14905-w)
Supplement: Supplementary file 1 — Supplementary Information [file 41467_2020_14905_MOESM1_ESM.pdf]

Supplementary Information

The epidermis coordinates thermoresponsive growth through the phyB-PIF4-auxin pathway

Kim et al.

a

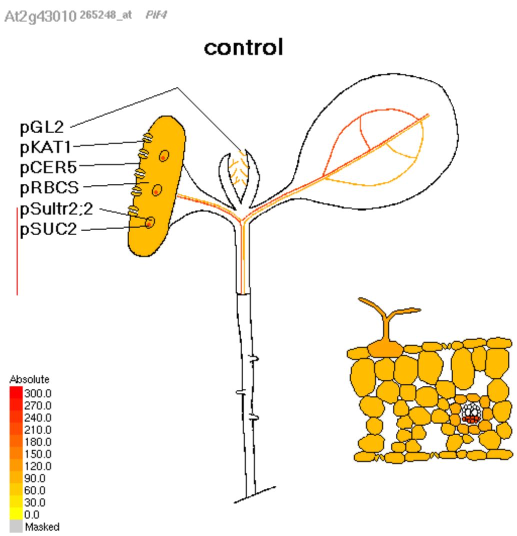

b

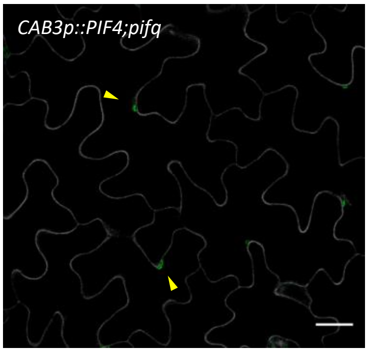

Supplementary Fig. 1 Tissue-specific expression of *PIF4*. a, *PIF4* expression in *Arabidopsis* according to the Cell-Type Specific Arabidopsis eFP browser (http://efp.ucr.edu/). **b,** A confocal microscopic image showing PIF4-YFP expression in the epidermis in *CAB3p::PIF4-YFP;pifq* transgenic plants. PI (gray) was used to counterstain the cell walls. Scale bars = 20 µm.

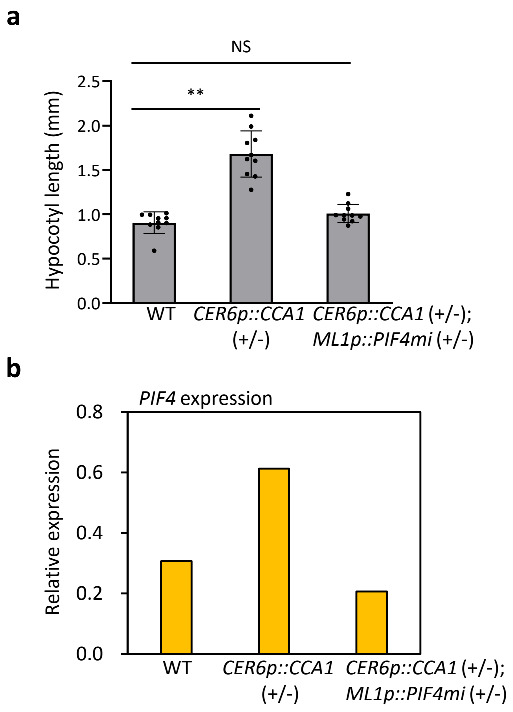

Supplementary Fig. 2 | The long hypocotyls of *CER6p::CCA1* plants were suppressed by down-regulation of *PIF4*. a, Hypocotyl lengths of seedlings grown under continuous white light at 20 °C for 7 days. Error bars indicate s.d. (n = 10 plants). ** $P < 0.01$ (two-tailed Student's *t*-test); NS, not significant (Student's *t*-test $P \geq 0.05$). **b,** *PIF4* expression levels in the seedlings grown in the same growth condition as (a). The expression levels of *PIF4* were normalized to those of *PP2A*.

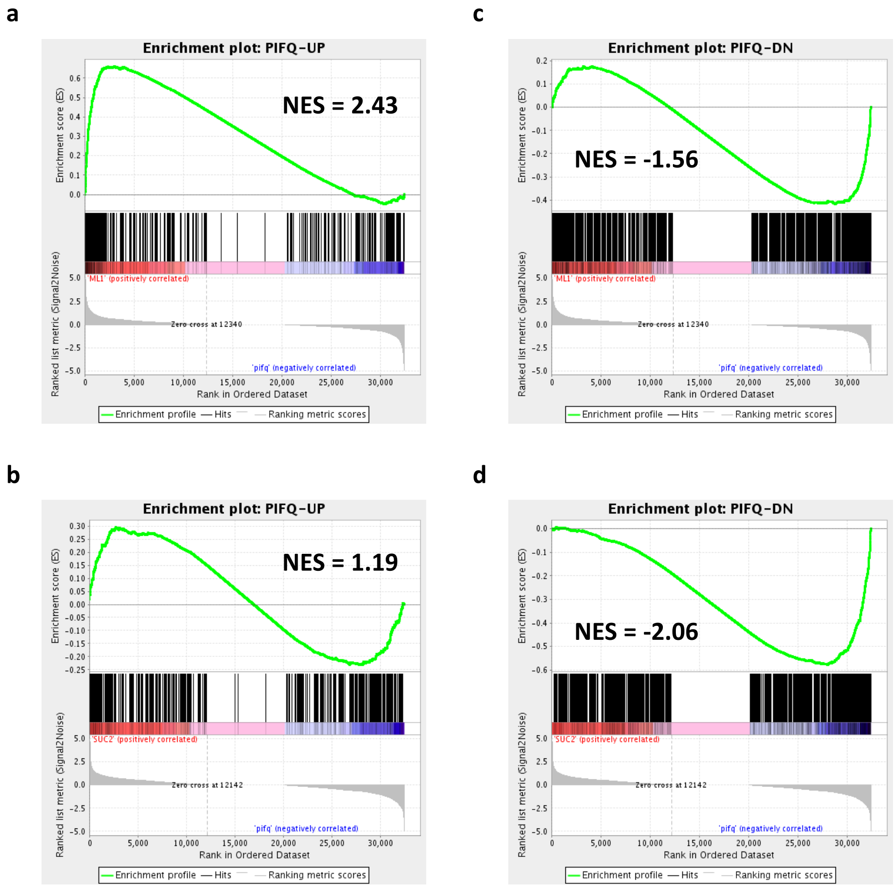

Supplementary Fig. 3 | Gene set enrichment analysis (GSEA) of global PIF-regulated genes by tissue-specific PIF4. The global PIF quartet-activated genes (PIFQ-UP) and -repressed genes (PIFQ-DN) were determined as differentially expressed genes in RNA-seq comparison of the wild type to *pifq* (1.5-fold, adjusted *P*-value < 0.05) (Fig. 2e, f). The enrichment of PIFQ-UP and PIF-DN gene sets were examined by GSEA in RNA-seq comparison of *ML1p::PIF4;pifq* to *pifq* (**a, c**) or *SUC2p::PIF4;pifq* to *pifq* (**b, d**), respectively. NES indicates a normalized enrichment score.

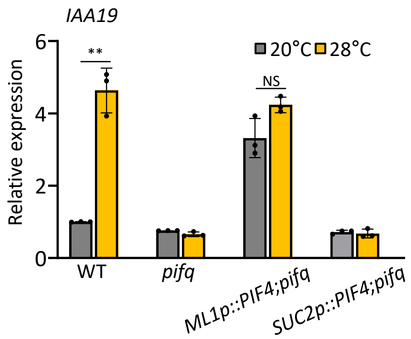

Supplementary Fig. 4 | qRT-PCR analyses of *IAA19*. WT and transgenic seedlings were grown in 12 h light/12 h dark cycles (12L:12D) at 20 °C for 4 days and transferred under the continuous white light on the 5th day. The growth temperature was increased to 28 °C or kept at 20 °C for 4 h at ZT20-24 before harvesting for total RNA extraction. Gene expression levels were normalized to *APX3* and presented as values relative to that of wild-type (WT) kept at 20 °C. Error bars indicate s.d. (n=3). **$P < 0.01$ (two-tailed Student's t-test). NS, not significant (two-tailed Student's t-test $P \geq 0.05$)

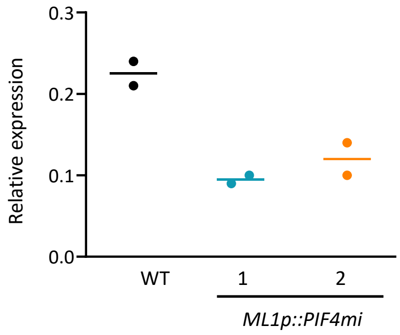

Supplementary Fig. 5 | *PIF4* mRNA expression levels in the wild type and *ML1p::PIF4mi* seedlings. Seedlings were grown under continuous white light at 20 °C for 5 days and harvested for RNA extraction. The gene expression levels were normalized to *PP2A*. Lines indicate median values ($n = 2$).

a

b

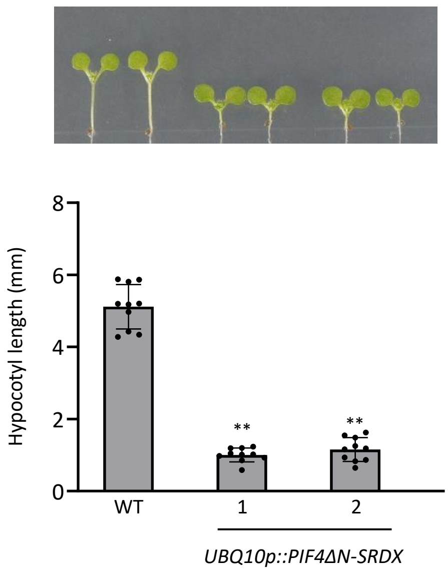

Supplementary Fig. 6 | PIF4ΔN-SRDX inhibits hypocotyl growth at high temperatures. Hypocotyl lengths of seedlings grown under continuous white light at 20 °C for 4 days followed by 28 °C for 3 days. Representative seedlings are shown in (**a**). Error bars in (**b**) indicate s.d. (n = 10 plants). **P < 0.01 (two-tailed Student's t-test).

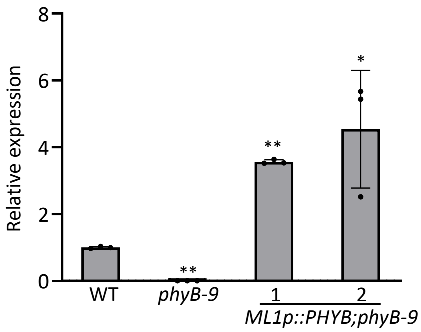

Supplementary Fig. 7 | The expression levels of *PHYB* in WT, *phyB-9*, and *ML1p::PHYB;phyB-9* seedlings. Seedlings were grown under continuous white light at 20 °C for 5 days and harvested for RNA extraction. The levels of *PHYB* were determined by qRT-PCR using the specific primers that amplify the wild-type *PHYB* cDNA, but not mutant *PHYB* cDNA in *phyB-9* (Supplementary Table 1). The gene expression levels were normalized to *APX3*. Error bars indicate s.d. ($n = 3$). **$P < 0.01$, *$P < 0.05$ (two-tailed Student's *t*-test).

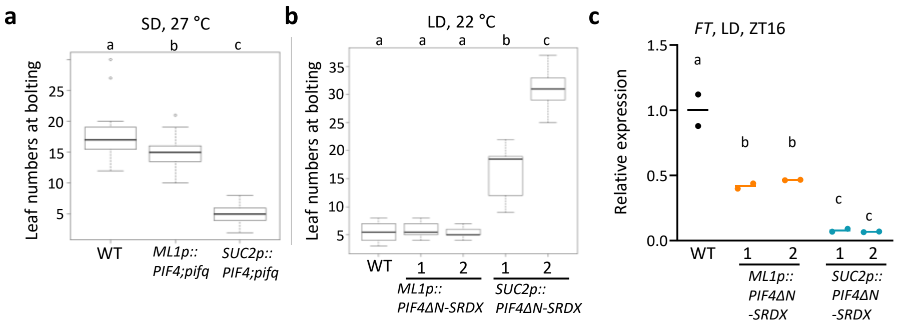

Supplementary Fig. 8 | Vascular PIF4 plays a dominant role in the regulation of flowering. a, Flowering time of WT, *ML1p::PIF4;pifq*, and *SUC2p::PIF4;pifq* plants grown under short days at 27 °C. Flowering time was scored as the number of rosette leaves at bolting. Letters above each box indicate significant differences based on a one-way ANOVA and Tukey's test ($P < 0.05$). In the box plots (**a**, **b**), the thick lines indicate median values, the lower and upper ends of the boxes represent the 25th and 75th percentiles, and the ends of the whiskers are set at 1.5 times the interquartile range. **b,** Flowering time of WT, *ML1p::PIF4ΔN-SRDX*, and *SUC2p::PIF4ΔN-SRDX* plants grown under long days at 22 °C. Flowering time was scored as the number of rosette leaves at bolting. Letters indicate significant differences based on a one-way ANOVA and Tukey's test ($P < 0.05$). **c,** *FT* mRNA expression of seedlings grown under long days at 22 °C. Seedlings were harvested at ZT16 for total RNA extraction. The *FT* expression levels were normalized to *APX3* and presented as values relative to that of WT. Lines indicate median values ($n = 2$). Letters above each bar indicate significant differences based on a one-way ANOVA and Tukey's test ($P < 0.05$).

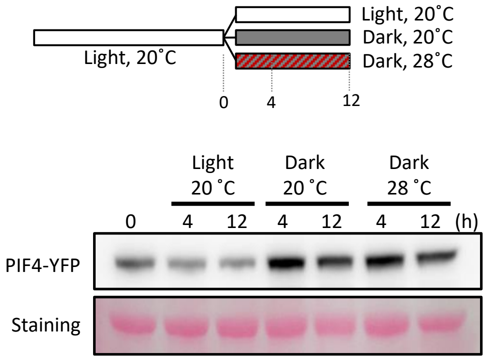

Supplementary Fig. 9 | Darkness increases the levels of epidermal PIF4 in *ML1p::PIF4;pifq*. The *ML1p::PIF4;pifq* seedlings grown under white light at 20 ˚C were kept under white (20 ˚C) or transferred to darkness (20 or 28 ˚C). The seedlings were harvested after 4 or 12 h for total protein extraction. Immunoblotting was probed using an anti-GFP antibody. Ponceau S staining is shown for equal loading.

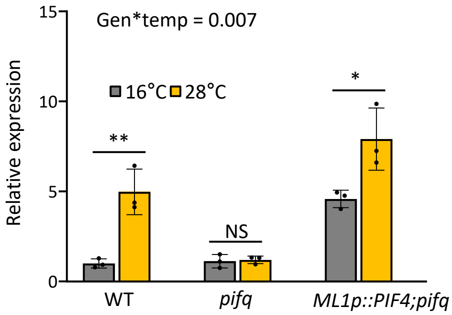

Supplementary Fig. 10 | qRT-PCR analyses of *YUC8*. WT, *pifq* and *ML1p::PIF4;pifq* seedlings were grown in 12 h light/12 h dark cycles (12L:12D) at 16 °C for 6 days and transferred under the continuous white light on the 7th day. The growth temperature was increased to 28 °C or kept at 16 °C for 4 h at ZT20-24 before harvesting for total RNA extraction. Gene expression levels were normalized to *APX3* and presented as values relative to that of wild-type (WT) kept at 20 °C. Error bars indicate s.d. (n = 3). The P-value for the interaction term (genotype x temperature) calculated by two-way ANOVA is shown at the top. ** $P < 0.01$, * $P < 0.05$ (two-tailed Student's t-test). NS, not significant (two-tailed Student's t-test $P \geq 0.05$).

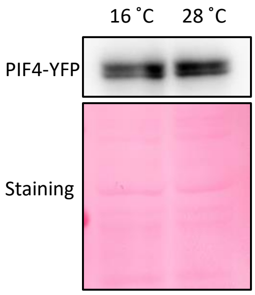

Supplementary Fig. 11 | High-temperature effects on the stability of epidermal PIF4 protein. *ML1p::PIF4-YFP;pifq* seedlings were grown under white light constitutively at 16 °C or grown at 16 °C and shifted to 28 °C for 24 h. Immunoblotting was probed using an anti-GFP antibody. Ponceau S staining is shown for equal loading.

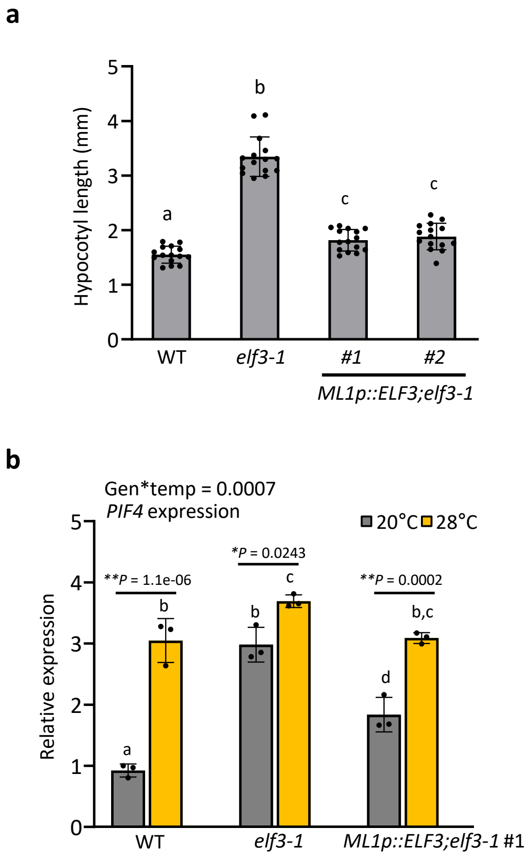

Supplementary Fig. 12 | Epidermal ELF3 represses both hypocotyl growth and the expression of *PIF4*. a, Hypocotyl lengths of seedlings grown under the continuous white light at 20 °C for 7 days. Error bars indicate s.d. (n = 15 plants). Different letters above the bars indicate significant differences based on one-way ANOVA and Tukey's test ($P < 0.05$). **b,** qRT-PCR analyses of *PIF4* mRNA expression. Seedlings were grown in 12 h light/12 h dark cycles (12L:12D) at 20 °C for 4 days and transferred under the continuous white light on the 5th day. The growth temperature was increased to 28 °C or kept at 20 °C for 4 h at ZT20-24 before harvesting for total RNA extraction. Gene expression levels were normalized to *APX3*. Error bars indicate s.d. (n = 3). The P-value for the interaction term (genotype x temperature) calculated by two-way ANOVA is shown at the top. Letters above the bars indicate significant differences based on Tukey's test ($P < 0.05$). Black asterisks indicate significant differences (**$P < 0.01$ and *$P < 0.05$, two-tailed Student's t-test).

qRT-PCR		
**Gene**	**Forward**	**Reverse**
PP2A	TATCGGATGACGATTCTTCGTGCAG	GCTTGGTCGACTATCGGAATGAGAG
APX3	AGAGCACACTCATGGTGCCAAC	TGCTTAGCTTTCACGCCCTCAC
PIF4	GCCAAAACCCGGTACAAAACCA	CGCCGGTGAACTAAATCTCAACATC
YUC8	AAACGCTCAAGGGGTTCTCTTCG	CACGCACAACACCCTTTGATTCG
IAA19	GGTGACAACTGCGAATACGTTACCA	CCCGGTAGCATCCGATCTTTTCA
IAA29	AAACAGCGTTTGTTTGCCTTGAATG	TGGCCATCCAACAACTTCGCTAT
PHYB	GAAGAAGCTCGATGAGGCTTAGG	AACTGTAAACCGAAAGCCTGCATC
ELF3	TTCATCCTGGACCATCTAGTCAGC	GTTGCTTGGTTTGCGGCTGAAG
***ML1* and *SUC2* promoters**		
	**Forward**	**Reverse**
ML1 promoter (CACC-BamHI)	CACCCAAGAACAAAACGATGCATAG	GGATCCTAACCGGTGGATTCAGGGAGTT
SUC2 promoter (CACC-BamHI)	CACCAAAATCTGGTTTCATATTAATTTCA	GGATCCATTTGACAAACCAAGAAAGTAAG
**ChIP-qPCR**		
**Gene**	**Forward**	**Reverse**
PP2A	CGGCTTTCATGATTCCCTCT	GCCTTAAGCTCCGTTTCCTACTT
UBC30	CAAATCCAAAACCCTAGAAACCGAA	AACGACGAAGATCAAGAACTGGGAA
rDNA	CCCAAGTCAGACGAACGATT	TCTGACATGTGTGCGAGTCA
YUC8	TGGTTCCACACAATTTTCACAG	GCAACGATGGTGATTGTTGAAG
IAA19	TAACCACCTTGTAATGCCGGTC	GCAGAGACAGGTCAACTGAGGA
ATHB2	ACAACAAAAACAGAAGGGGTAGT	CCTTAAGCTAATCGACAAGTCAA

Supplementary Table. 1 | Primer list for qRT-PCR, ChIP-qPCR, and vector construction
