# Supplementary material for: The epidermis coordinates thermoresponsive growth through the phyB-PIF4-auxin pathway
Source: Nat Commun. 2020 Feb 26;11:1053. doi: 10.1038/s41467-020-14905-w (PMC7044213; doi:10.1038/s41467-020-14905-w)
Supplement: Supplementary file 3 — Description of Additional Supplementary Files [file 41467_2020_14905_MOESM3_ESM.pdf]

Description of Additional Supplementary Files

File Name: Supplementary Data 1
Description: List of tissue-specific PIF4-regulated genes

File Name: Supplementary Data 2
Description: List of epidermal or vascular PIF4-regulated genes and auxin-regulated genes

File Name: Supplementary Data 3
Description: Comparison of epidermal or vascular PIF4-regulated genes with PIF4 direct target genes
