# Supplementary material for: The epidermis coordinates thermoresponsive growth through the phyB-PIF4-auxin pathway
Source: Nat Commun. 2020 Feb 26;11:1053. doi: 10.1038/s41467-020-14905-w (PMC7044213; doi:10.1038/s41467-020-14905-w)
Supplement: Supplementary file 7 — Reporting Summary [file 41467_2020_14905_MOESM7_ESM.pdf]

nature research

Corresponding author(s): Eunkyoo Oh

Last updated by author(s): Jan 28, 2020

Reporting Summary

Nature Research wishes to improve the reproducibility of the work that we publish. This form provides structure for consistency and transparency in reporting. For further information on Nature Research policies, see Authors & Referees and the Editorial Policy Checklist .

Statistics

For all statistical analyses, confirm that the following items are present in the figure legend, table legend, main text, or Methods section.

n/a	Confirmed	
☐	☒	The exact sample size (*n*) for each experimental group/condition, given as a discrete number and unit of measurement
☐	☒	A statement on whether measurements were taken from distinct samples or whether the same sample was measured repeatedly
☐	☒	The statistical test(s) used AND whether they are one- or two-sided<br>*Only common tests should be described solely by name; describe more complex techniques in the Methods section.*
☒	☐	A description of all covariates tested
☒	☐	A description of any assumptions or corrections, such as tests of normality and adjustment for multiple comparisons
☐	☒	A full description of the statistical parameters including central tendency (e.g. means) or other basic estimates (e.g. regression coefficient) AND variation (e.g. standard deviation) or associated estimates of uncertainty (e.g. confidence intervals)
☐	☒	For null hypothesis testing, the test statistic (e.g. *F*, *t*, *r*) with confidence intervals, effect sizes, degrees of freedom and *P* value noted<br>*Give P values as exact values whenever suitable.*
☒	☐	For Bayesian analysis, information on the choice of priors and Markov chain Monte Carlo settings
☒	☐	For hierarchical and complex designs, identification of the appropriate level for tests and full reporting of outcomes
☒	☐	Estimates of effect sizes (e.g. Cohen's *d*, Pearson's *r*), indicating how they were calculated

Our web collection on statistics for biologists contains articles on many of the points above.

Software and code

Policy information about availability of computer code

Data collection	No software was used.
Data analysis	WMD3 was used to design microRNA. Salmon (1.2.0) and DESeq2 software was used to analyze RNA-Seq data. ImageJ (1.52s) software was used to measure the hypocotyl lengths of seedlings.

For manuscripts utilizing custom algorithms or software that are central to the research but not yet described in published literature, software must be made available to editors/reviewers. We strongly encourage code deposition in a community repository (e.g. GitHub). See the Nature Research guidelines for submitting code & software for further information.

Data

Policy information about availability of data

All manuscripts must include a data availability statement. This statement should provide the following information, where applicable:

- Accession codes, unique identifiers, or web links for publicly available datasets
- A list of figures that have associated raw data
- A description of any restrictions on data availability

RNA sequencing data were deposited into the Gene Expression Omnibus database (accession number GSE133564). Arabidopsis mutants and transgenic lines used in this study are available upon request from the corresponding author. The source data for Figs. 2a-d, 3a-d, 3f-h, 4a-d, 5a, 6a-c and 6f-i and Supplementary Figs. 2, 4, 6, 7, and 9-11 are provided as a Source Data file.

Field-specific reporting

Please select the one below that is the best fit for your research. If you are not sure, read the appropriate sections before making your selection.

Life sciences study design

All studies must disclose on these points even when the disclosure is negative.

Sample size	No statistical methods were used to predetermine sample sizes. Sample sizes were chosen based on previous studies and reproducibility.
Data exclusions	No data were excluded.
Replication	At least 10 seedlings were used for hypocotyl measurements. The experiments were repeated three times with similar results. Three biological replicates (each replicate contains about 50 seedlings for transcript analyses/immunoblots and 500 seedlings for ChIP assays) were performed for transcript analyses, ChIP assays, and immunoblots. All attempts at replication was successful.
Randomization	For hypocotyl measurements, transcript analyses, and immunoblots, the seedlings were exposed to light uniformly and were randomly selected for the analyses.
Blinding	Not relevant to this study, because the phenotypes of the mutants are obvious.

Reporting for specific materials, systems and methods

We require information from authors about some types of materials, experimental systems and methods used in many studies. Here, indicate whether each material, system or method listed is relevant to your study. If you are not sure if a list item applies to your research, read the appropriate section before selecting a response.

Materials & experimental systems

n/a	Involved in the study	
☐	☒	Antibodies
☒	☐	Eukaryotic cell lines
☒	☐	Palaeontology
☒	☐	Animals and other organisms
☒	☐	Human research participants
☒	☐	Clinical data

Methods

n/a	Involved in the study	
☒	☐	ChIP-seq
☒	☐	Flow cytometry
☒	☐	MRI-based neuroimaging

Antibodies

Antibodies used	Anti-Myc antibody, Cell signaling, 2276S; Anti-GFP antibody, Thermo Fisher Scientific, A-11122
Validation	The validation data for anti-Myc antibody and anti-GFP antibody can be found in the manufacturer's website.<br>https://media.cellsignal.com/pdf/2276.pdf<br>https://www.thermofisher.com/order/genome-database/dataSheetPdf?<br>producttype=antibody&productsubtype=antibody_primary&productId=A-11122&version=89
